# Strengths and limitations of the use of Artificial Intelligence in Mental Health

**DOI:** 10.1192/j.eurpsy.2025.1426

**Published:** 2025-08-26

**Authors:** A. Alberca-González, E. Fernández-Jiménez

**Affiliations:** 1 Faculty of Social Sciences and Communication, Universidad Europea de Madrid, Villaviciosa de Odón; 2 Department of Psychiatry, Clinical Psychology and Mental Health, La Paz University Hospital; 3 Hospital La Paz Institute for Health Research (IdiPAZ), Madrid, Spain

## Abstract

**Introduction:**

Artificial intelligence (AI) is emerging as a disruptive tool in medicine and healthcare, especially in Mental Health. Its ability to process large volumes of data and detect complex patterns has opened up new possibilities for the diagnosis, treatment, and monitoring of mental disorders, providing opportunities to improve the clinical accuracy and personalisation of therapeutic intervention. However, the use of AI in Mental Health poses critical challenges involving ethical, privacy, and inherent issues regarding the quality and validity of the models employed.

**Objectives:**

The aim of this review is to critically analyse the strengths and limitations of the use of AI in Mental Health, offering a balanced perspective on the potential benefits and associated risks.

**Methods:**

A narrative review of the current scientific literature was conducted on the main applications of AI in the diagnosis, treatment, and monitoring of mental disorders as well as the barriers to its effective implementation.

**Results:**

The main strengths identified were the ability of AI to provide more accurate and personalised diagnoses, continuous monitoring of patients’ well-being, increased efficiency in the delivery of Mental Health services, and the possibility of analysing large volumes of data in reduced times, thus improving the ability to detect and track symptoms. However, regarding limitations, the scientific literature highlights the lack of transparency in many of the studies conducted, problems of methodological quality, risks regarding the perpetuation of pre-existing biases, concerns about the privacy of sensitive data, and the potential risk of dehumanisation of care by prioritising automated systems over human contact. In addition, the need for rigorous validation and a clear regulatory framework are key issues in ensuring the ethical and safe use of these technologies.

**Conclusions:**

In conclusion, AI represents a resource with immense potential for transforming clinical practice in Mental Health care, providing more accurate diagnoses, personalised interventions, and real-time monitoring. However, its implementation must be accompanied by rigorous scientific and regulatory scrutiny to mitigate ethical risks and ensure the protection of patients’ privacy and dignity. It is necessary to ensure that technological advances go hand in hand with a humanised approach, where technology complements, but does not replace, the essential therapeutic connection in Mental Health care.

**Disclosure of Interest:**

None Declared

